# Prognostic value of presepsin in adult patients with sepsis: Systematic review and meta-analysis

**DOI:** 10.1371/journal.pone.0191486

**Published:** 2018-01-24

**Authors:** Hyun Suk Yang, Mina Hur, Ahram Yi, Hanah Kim, Seungho Lee, Soo-Nyung Kim

**Affiliations:** 1 Department of Cardiovascular Medicine, Research Institute of Medical Science, Konkuk University School of Medicine, Seoul, Korea; 2 Department of Laboratory Medicine, Konkuk University School of Medicine, Seoul, Korea; 3 School of Public Health, Seoul National University, Seoul, Korea; 4 Department of Obstetrics and Gynecology, Konkuk University School of Medicine, Seoul, Korea; Azienda Ospedaliero Universitaria Careggi, ITALY

## Abstract

**Objective:**

Presepsin is a novel biomarker to diagnose sepsis but its prognostic value has not been comprehensively reviewed. We conducted this meta-analysis to evaluate the mortality prediction value of presepsin in sepsis.

**Methods:**

We searched comprehensive electronic databases from PubMed, EMBASE, and Cochrane Library through September 2017 using the key words of (‘presepsin’ or ‘sCD14-ST’ or ‘soluble CD14 subtype’) and (‘sepsis’ or ‘septic shock’) and (‘prognosis’ or ‘prognostic value’ or ‘prognostic biomarker’ or ‘mortality’). We extracted the presepsin levels in survivors and non-survivors from each individual study and evaluated the standardized mean difference (SMD) using a web-based meta-analysis with the R statistical analysis program.

**Results:**

A total of 10 studies and 1617 patients were included. Presepsin levels in the first sampling (within 24 hours) were significantly lower among survivors as compared with non-survivors: the pooled SMD between survivors and non-survivors was 0.92 (95% CI: 0.62–1.22) in the random effects model (*I*^2^ = 79%, *P*< 0.01). In subgroups, divided by the sepsis severity or study site, pooled SMD was consistently noting higher presepsin levels in non-survivals (*P*< 0.05).

**Conclusion:**

This meta-analysis demonstrates some mortality prediction value in presepsin in patients with sepsis. Further studies are needed to define the optimal cut-off point to predict mortality in sepsis.

## Introduction

Sepsis is defined as life-threatening organ dysfunction caused by a dysregulated host response to infection [1]. It’s still a clinically challenging syndrome with a mortality range of 10% to 52% [2–4]. To promptly recognize and manage higher risk patients, several risk stratification models have been adopted such as Sequential Organ Failure Assessment (SOFA) [5], Acute Physiology And Chronic Health Evaluation (APACHE) [6]or Mortality in Emergency Department Sepsis (MEDS) [7] scores. For still more robust prognostication, multiple biomarkers have been suggested including presepsin.

Presepsin, also known as soluble CD14 subtype, is a 13-kDa glycoprotein cleavage N-terminal fragment of CD14, released into circulation after activation of a pro-inflammatory signal cascade on contact with infectious agents [8]. Presepsin can be detected by biochemical methods and has been considered an emergent biomarker of infection. In 2002, presepsin was first discovered as a blood biomarker in patients with sepsis in Japan [9]. In 2015, its diagnostic accuracy in sepsis was confirmed by meta-analysis [10–12], but the prognostic accuracy of presepsin in sepsis was only reported in individual clinical studies, some showing significantly lower early presepsin levels in survivors compared with non-survivors [13–21], others not [22, 23].

Therefore, we conducted a comprehensive systematic review and meta-analysis to evaluate the mortality prediction value of presepsin in adult patients with sepsis.

## Methods

### Literature search

We performed a comprehensive electronic search of PubMed, EMBASE, and the Cochrane Library without language limitations through September 2017. The search terms used were: (‘presepsin’ or ‘sCD14-ST’ or ‘soluble CD14 subtype’) and (‘sepsis’ or ‘septic shock’) and (‘prognosis’ or ‘prognostic value’ or ‘prognostic biomarker’ or ‘mortality’). References from relevant articles were also reviewed.

### Study selection

A study was eligible for this meta-analysis if it was a clinical study conducted in patients suffering from sepsis (including severe sepsis and septic shock) according to the international sepsis definition [1, 24–27], and showed the presepsin levels in survivors and non-survivors within 24 hours of the diagnosis of sepsis. Published abstracts were also reviewed if they carried the pertinent information. The studies considered ineligible for this meta-analysis were review articles, editorials, case reports, studies on pediatrics, and studies with insufficient information to discern the mean and standard deviation of presepsin levels. No restrictions have been applied regarding the study setting or comorbidities for data collection. In case of multiple publications with the same or over-lapping cohort, only the published report with the largest series was included. All data selections were completed by two reviewers (HSY, MH) independently, and any discrepancies were resolved by consensus discussion or consulting a third reviewer (SNK).

### Data extraction

We extracted the following data from each eligible study: year of publication, site of study, severity of sepsis, time of first sampling, definition of non-survivor, and presepsin levels in survivors and non-survivors (expressed as mean and SD or median and range). In case of multiple presepsin sampling within 24 hours, we chose the first sampling result for the meta-analysis. If the presepsin levels were provided as median and range, the mean and SD were estimated by Hozo et al’s method [28], or estimated as mean = (2*m* + *a* + *b*)/4, where *m* is the median and *a* and *b* are the 25^th^ and 75^th^ percentiles, as SD = IQR/1.35 by the Cochrane handbook formula [29], unless we were able to obtain additional data information from the original authors.

### Quality assessment

Studies were evaluated for methodological quality utilizing elements from the QUADAS 2 checklist [30], systematically noting 4 criteria: whether (1) the study included consecutive patients (selection bias), (2) the professionals who influenced the outcomes were blinded to the presepsin result at study entry (confusion bias), (3) the timing of blood sample was within 24 hours after diagnosis (information bias), and (4) the study excluded comorbidities potentially influencing presepsin levels and accuracy (confounding bias). Each of these four criteria was evaluated independently by two reviewers (HSY, MH), then any disagreement was resolved after discussion and reevaluation by a third reviewer (SNK).

### Statistical analysis

We performed statistical analysis using web-based meta-analysis with R (http://web-r.org). Heterogeneity was explored using the statistic *I*^2^: a significant heterogeneity exists when *I*^2^>56%. The pooled standardized mean difference (SMD) and the 95% confidence interval (95% CI) were calculated using the random effects model. Pooled SMD was considered significant if *P* < 0.05. We performed subgroup analyses to explore the prognostic value of presepsin in different clinical settings such as in the intensive care unit (ICU) or in the emergency department (ED), and with different sepsis severities, as post hoc analyses. The publication bias was explored using the Eggar test via a funnel plot, with *P*< 0.05 indicating a significant bias.

## Results

### Characteristics of included studies

**Fig 1:**
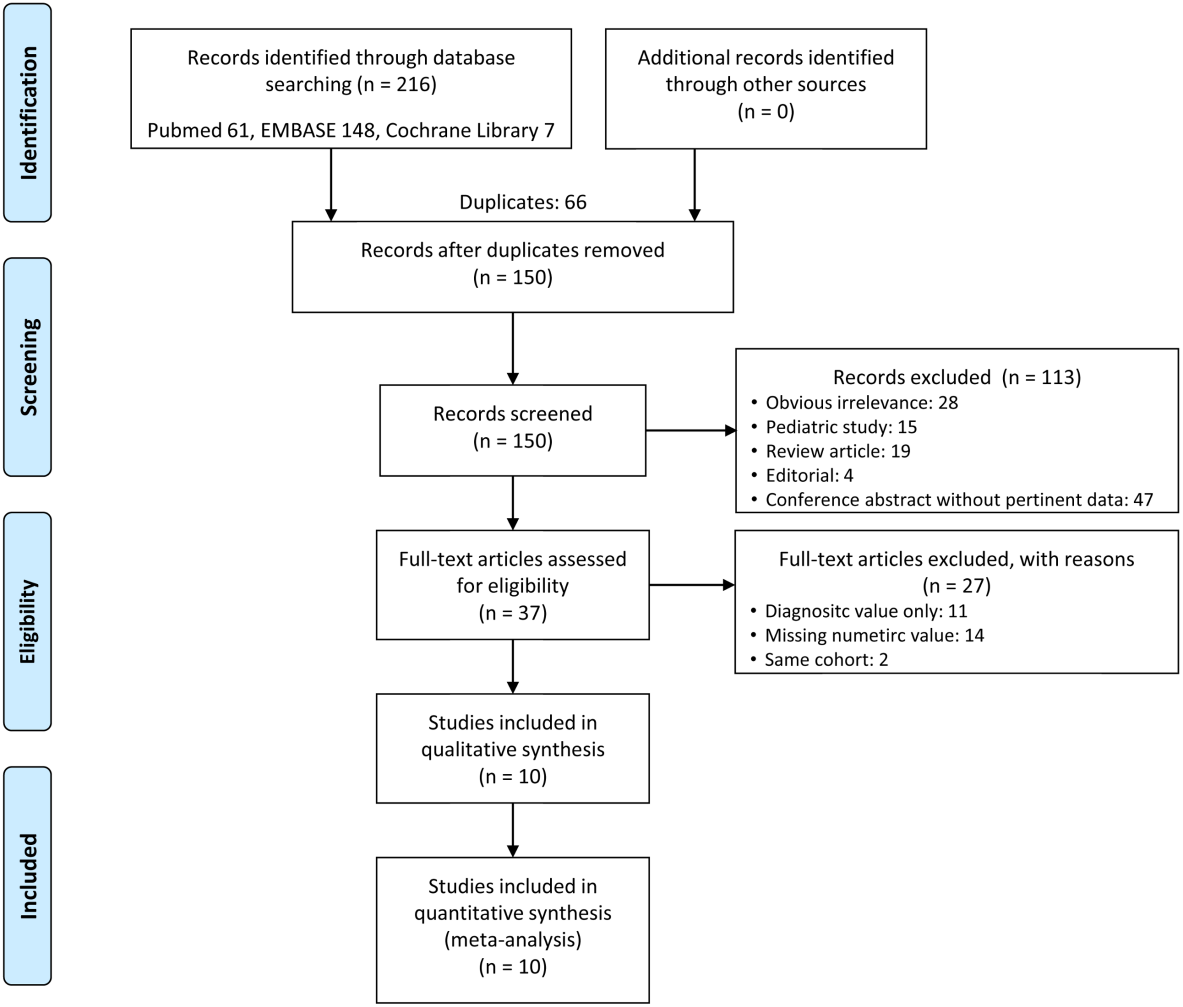
A flow diagram of the study selection process.

A total 10 studies with 1617 patients were included in this systematic review and meta-analysis followed by the systematic selection flow diagram (Fig 1). The characteristics of included studies are in Table 1. Six studies were prospectively observational [13, 17–20, 22], while three were retrospective cohort studies from other prospectively-collected registries (Mannheim sepsis study [14], Surviving Sepsis Campaign 2012 [21], Original cohort of sepsis in University Hospital Brno [16]), and one was a retrospective case—control study from the multi-center Albumin Italian Outcome Sepsis trial [15]. The publications ranged in date from 2013 to 2017; actual study recruitment time varied from October 2011 [14] to June 2015 [21]. Four studies were conducted in Europe [14–16, 19], three in Eastern Asia [13, 21, 22], two in Northwest Africa [18, 20], and one in South America [17]. All except one study were written in English; it was written in Czech [16] but contains an English abstract and table, and ultimately the full text was reviewed after English translation. Five studies were performed in the ICU [14, 15, 18–20], three in the ED [13, 17, 22], one in both the ICU and ED (60%, 30% respectively) [21], and one was in-hospital without specifically mentioning the ICU or ED [16].

**Table 1:** Characteristics of included studies.

Study	Type	Country, Site	Patients (n)	Age	Male (%)	Infection source (%)	Sepsis Severity	SOFA adm	APACHE II, adm	First sample	Definition, non-survivors	Non-survivors (%)	Quality score (/4)
**Liu et al**. 2013 [13]	Prospective	China, ED	859	71 (59–78)	60	lung (63), abdomen (22), meningitis (3), urinary (3), skin (1)	SIRS, S, SS, SSh	NR	16.0±6.8	≤24h	28 days	34	3
**Behnes et al**. 2014 [14]	Retrospective	Germany, ICU	74	68 (26–88)	70	lung (55), abdomen (16), urinary (5), skin (4), blood (10), others (10)	SSh	11.8±3.4	27±8.6	≤24h	6 month	72	3
**Masson et al**. 2014 [15]	Retrospective	Italy, ICU	100	71.5±12.3	54	abdomen (46), lung (38), urinary (18)	SS, SSh	8.5±2.9	NR	D1	ICU	50	1
**Beňovská et al**. 2015 [16]	Retrospective	Czech, adm	31	58.6 (50.8–62.7)	65	NA	S	NR	NR	D1	In-hospital	26	2
**Carpio et al**. 2015 [17]	Prospective	Peru, ED	123	67 (21–95)	46	urinary (34), lung (29), abdomen (25), catheter (4), skin (4), others (4)	SIRS, S, SS, SSh	NR	13.0±0.8	Adm	30 days	20	2
**Ali et al**. 2016 [18]	Prospective	Egypt, ICU	33	55.2±4.6	70	NR	^a^S	NR	16.1±4.6	Adm	28 days	58	1
**Klouche et al**. 2016 [19]	Prospective	France, ICU	100	58.3±16	61	lung (58), abdomen (11), meningitis (8), urinary (6)	SS, SSh	8.3±3.7	NR	Adm	ICU	25	4
**El-Shafie et al**. 2017 [20]	Prospective	Egypt, ICU	31	60 (52, 69)	52	NR	SIRS, S	7.0±2.9	20±9.63	Adm	in-hospital	36	3
**Kim et al**. 2017 [21]	Retrospective	Korea, ICU, ED	157	70 (58–77)	61	lung (65), urinary (35), abdomen (17)	^a^S, SSh	4.2±2.3	NR	Adm	30 days	22	2
**Yu et al**. 2017 [22]	Prospective	China, ED	109	74 (58.5, 82)	63	lung (58), abdomen (31), urinary (5), others (6)	SS	8.8±3.7	18.6±6.3	D1	90 days	60	2

Data presented as mean±SD, median (range), or median (Q1, Q3). Abbreviations: ED, emergency department; ICU, intensive care unit; adm, admission; D1, day one; SIRS, systemic inflammatory response syndrome with suspicious sepsis; S, sepsis; SS, severe sepsis; SSh, septic shock; NR, not reported; ^a^Sepsis defined on Sepsis-3 definition [1]

In Table 1, the included patients’ quantities and severities (sepsis severity, SOFA score, APACHE II score) are based on the patients analyzed as survivors or non-survivors; therefore, in 4 studies [14, 16, 18, 19] those numbers are different from the original study numbers. In all studies, identification of infection was performed on the bases of clinical features, laboratory findings, microbiological evidence and imaging tests [13–22]: some studies explicitly included patients with either proven or suspected infection [13, 17, 20], and one accepted only those with a blood culture positive infection [18]. The source of sepsis was explored in 7 studies [13, 14, 15, 17, 19, 21, 22], including 1504 patients: pulmonary in 58.6%, abdominal in 23.3%, urinary in 10.0%, meningitis in 2.0%, catheter-related in 0.8%, skin in 0.8%, others in 3.9% and unknown in 0.4%. In all 10 studies, presepsin measurements were performed with the PATHFAST system (Mitsubish Chemical or LSI Medience Corporation, Tokyo Japan) based on a chemi-luminescent enzyme immunoassay.

### Study quality and publication bias

**Fig 2:**
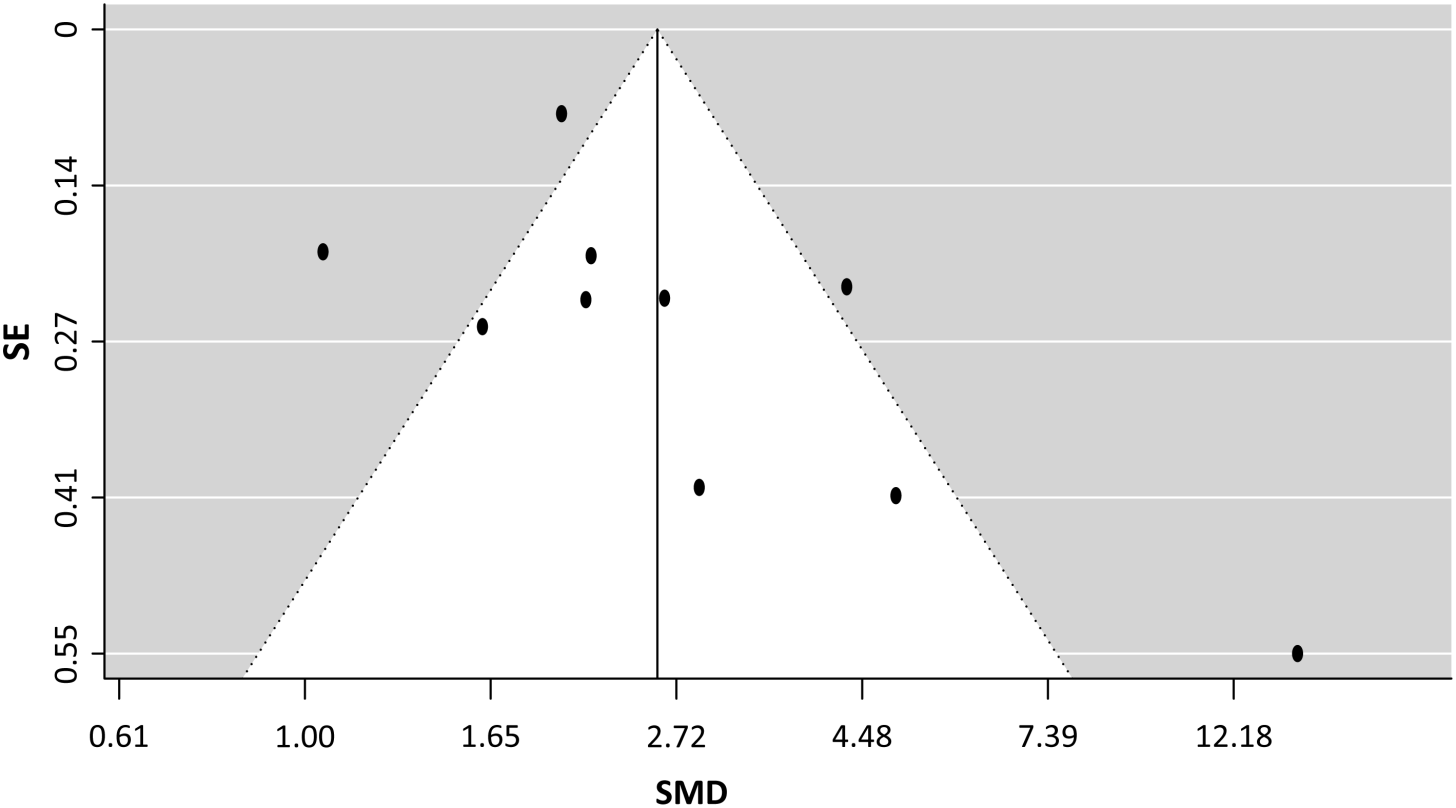
A funnel plot of the included studies. The Egger’s regression test with 95% confidence limits, Random-effect model, SMD (Standardized mean difference), SE (Standard error).

The total methodological quality scores by 4 criteria are presented in Table 1. All studies performed blood sampling within 24 hours or in the first day of diagnosis. Four studies [13, 17, 19, 21] enrolled consecutive patients to avoid selection bias; one retrospective case-control study [15] had inherent selection bias. Three studies [14, 19, 20] mentioned the blinding of professionals who influenced the outcome. Six studies [13, 14, 16, 19, 20, 22] excluded patients with comorbidities likely to influence presepsin levels such as terminal stage liver or kidney disease or traumatic or post-operative status. The funnel plot showed a trend that smaller studies are associated with larger effects; smaller studies not showing a significant effect may be less likely to be published. Even so, the linear regression test of funnel plot asymmetry demonstrates no significant publication bias (Fig 2) (*P* = 0.175).

### Meta-analysis

**Fig 3:**
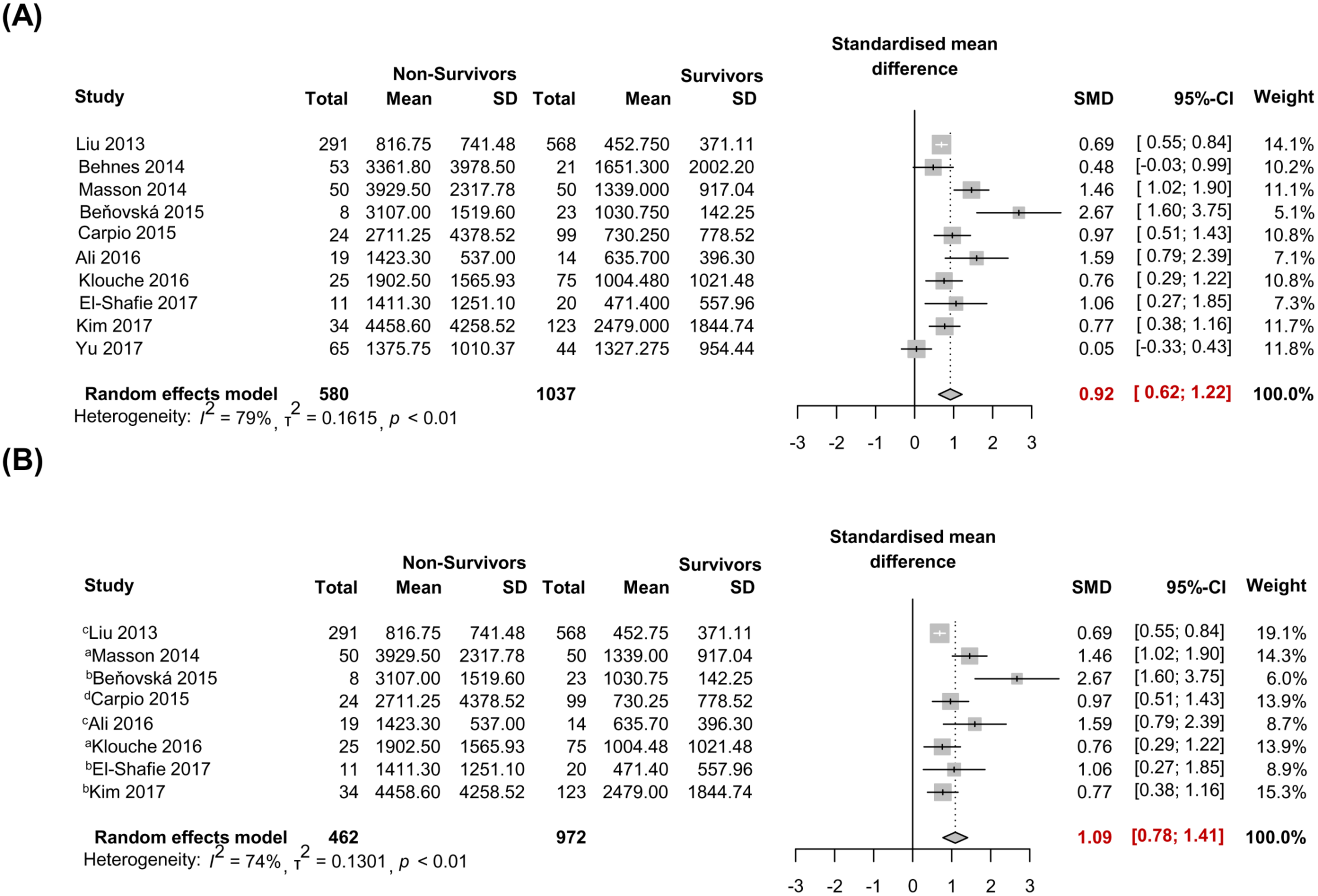
Forest plot of presepsin levels to predict mortality in sepsis. (A) Overall mortality with the 10 included studies. (B) Mortality with the short-term follow-up interval studies (^a^ intensive care unit, ^b^ In-hospital, ^c^ 28-days, ^d^ 30-days).

In this meta-analysis, 1617 patients from 10 studies were analyzed, with 580 non-survivors and 1037 survivors. The weighted pooled SMD of the first sampling of presepsin between non-survivors and survivors was 0.92 (0.62–1.22) by the random effects model with significant heterogeneity (*I*^2^ = 79%, *P*< 0.01) (Fig 3A); for the short-term follow-up interval studies (ICU, in-hospital, 28 or 30 day mortality) [13, 15–21], it was 1.09 (0.78–1.41) by the random effects model (*I*^2^ = 74%, *P*< 0.01) (Fig 3B).

#### Subgroup meta-analysis by sepsis severity

**Fig 4:**
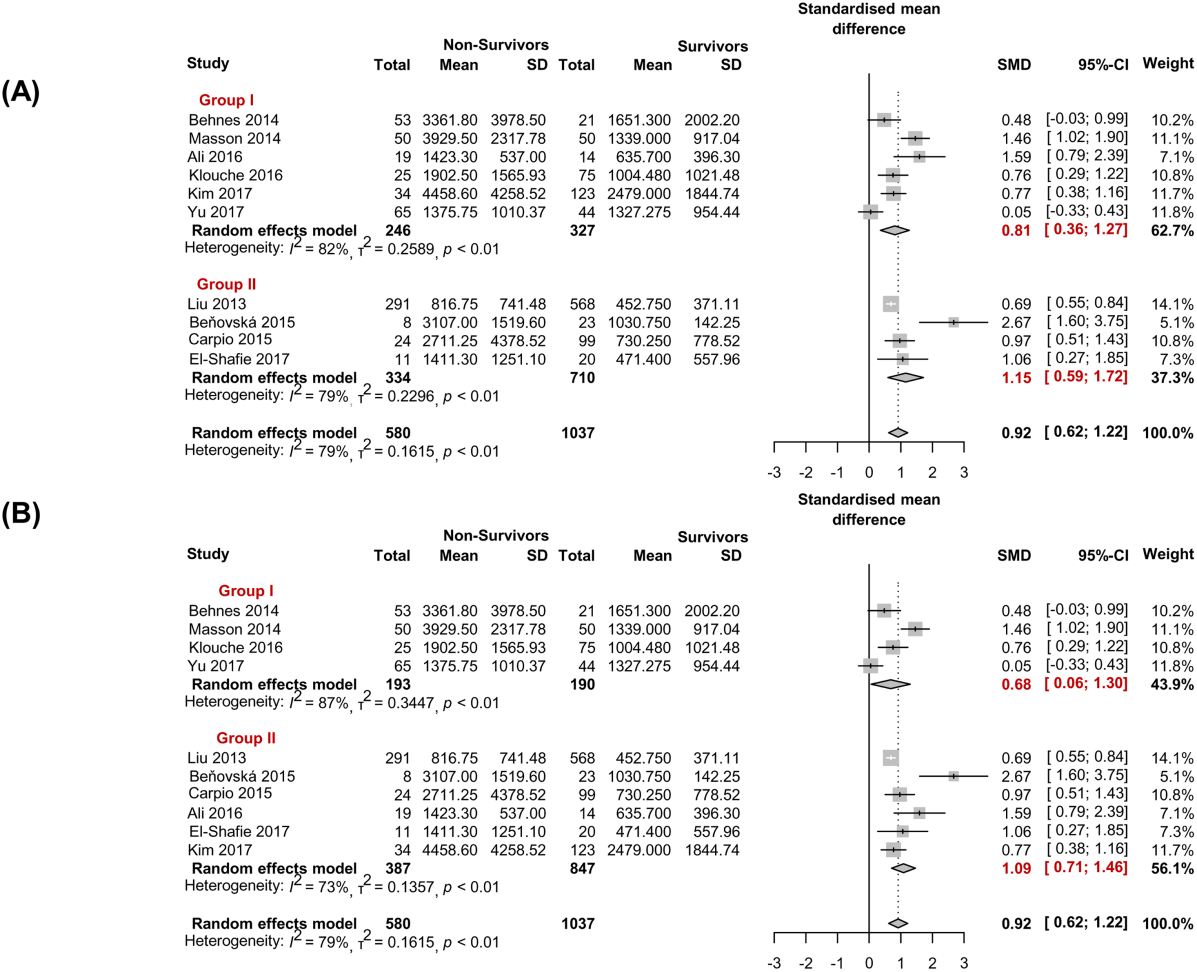
Subgroup analyses investigating presepsin to predict sepsis mortality according to the sepsis severity. (A) Studies with patient selection limited to severe sepsis and septic shock (Group I, n = 6) and studies without such a limitation (Group II, n = 4), (B) Studies with SOFA score≥8 (Group I, n = 4) and studies with SOFA score <8 or not reported (Group II, n = 6).

Six studies [14, 15, 18, 19, 21, 22] exclusively contain severe sepsis or septic shock (which means prior severe sepsis or septic shock, or the 3rd international consensus definition of sepsis and septic shock [1]). The pooled SMD between the non-survivors (n = 246) and survivors (n = 327) was 0.81 (0.36–1.27) by the random effects model (*I*^2^ = 82%, *P*< 0.01) (Fig 4A). In the studies with a SOFA score ≥8 [14, 15, 19, 22], the pooled SMD between the non-survivors (n = 193) and survivors (n = 190) was 0.81 (0.36–1.27) by the random effects model (*I*^2^ = 79%, *P*< 0.01) (Fig 4B).

#### Subgroup meta-analysis by study site

**Fig 5:**
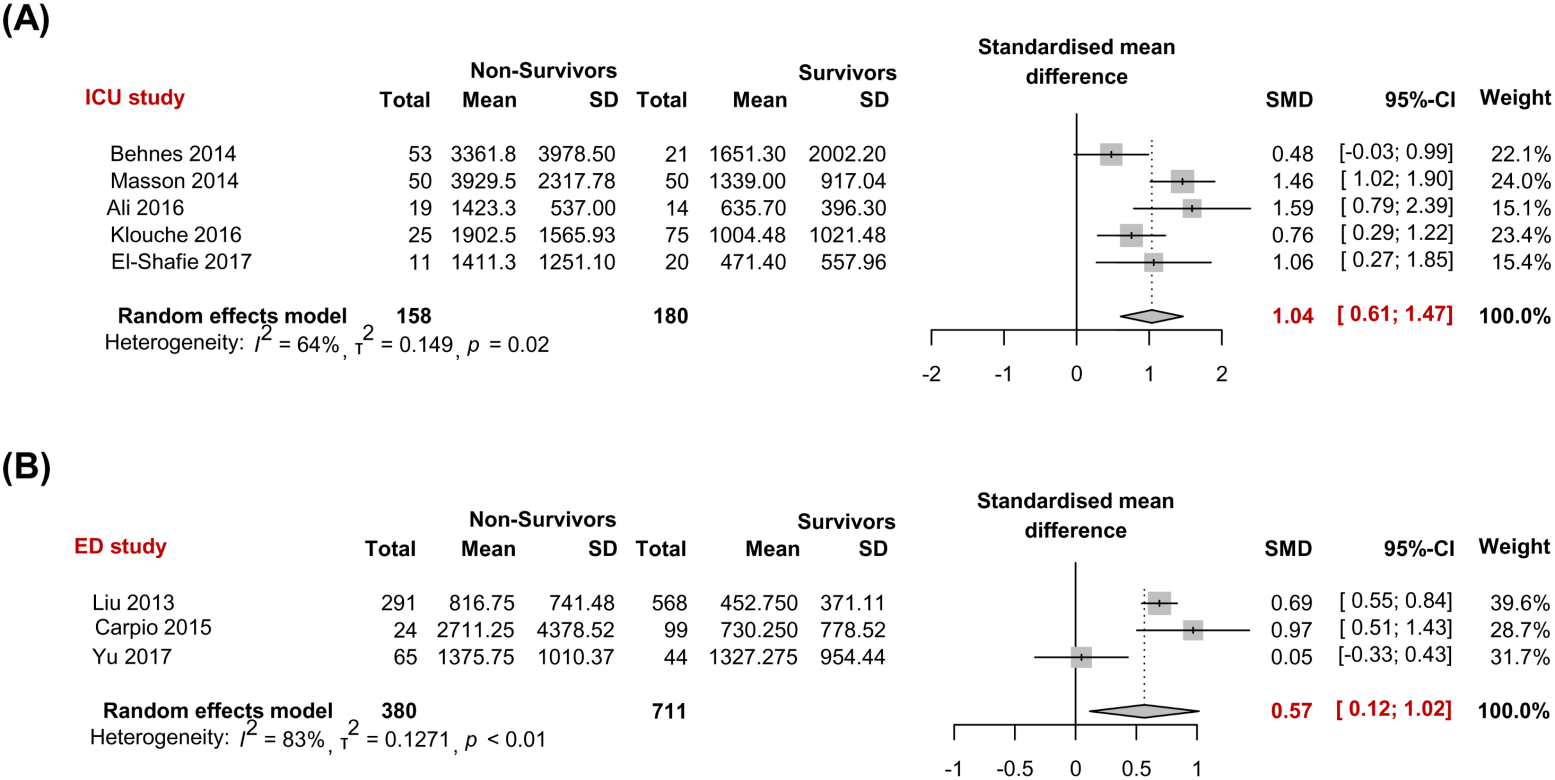
Subgroup analyses investigating presepsin to predict sepsis mortality according to the study location. (A) Studies conducted in an intensive care unit (ICU) or (B) emergency department (ED).

In 5 studies conducted in the ICU [14, 15, 18–20], the pooled SMD between the non-survivor (n = 158) and survivor (n = 180) was 1.04 (0.61–1.47) (*I*^2^ = 64%, P = 0.02); in 3 studies conducted in the ED [13, 17, 22] the pooled SMD between two was 0.57 (0.12–1.02) by the random effects model (*I*^2^ = 82%, *P*< 0.01) (Fig 5).

### Prediction of mortality

Seven studies [13–15, 17, 18, 20, 21] presented a receiver operating characteristic (ROC) analysis for the ability to use presepsin in prediction of mortality, but only 6 studies [13, 15, 17, 18, 20, 21] provided the cutoff values with sensitivity and specificity (Table 2).

**Table 2:** Prediction of mortality according to the first sampling (≤ 24 hours) of presepsin.

Study	Mortality (%)	AUC (95% CI)	Cutoff, ng/L	Sensitivity, %	Specificity, %	PPV, %	NPV, %
Liu 2013 [13]	28 d (34)	0.658 (0.614–0.703)	**556**	62.2	66.8	48.3	78.0
Behnes 2014 [14]	30 d (50)	0.64 (0.54–0.75)					
Masson 2014 [15]	ICU (57)	0.72 (0.61–0.82)	**1631**	70.2	67.4	74.1	63.0
Carpio 2015 [17]	30 d (19)	0.743	**825**	86	65	37.3	95.0
Ali 2016 [18]	28 d (58)	0.891 (0.765–1.000)	**957.5**	94.7	85.7	90.0	92.3
El-Shafie 2017 [20]	IH (36)	0.755 (0.938–0.571)					
Kim 2017 [21]	30 d (19)	0.684 (0.605–0.756)	**2455**	76.5	53.7	31.3	89.2

Abbreviations: d, days; ICU, intensive care unit; IH, in-hospital; CI, confidence interval; PPV positive predictive value; NPV, negative predictive value.

## Discussion

This is the first meta-analysis, to our knowledge, to demonstrate the mortality prediction value of presepsin in sepsis. The first-day presepsin levels were significantly higher in non-survivors as compared with survivors: a weighted pooled SMD of 0.92 (0.62–1.22) for over-all mortality and 1.09 (0.78–1.41) for in-hospital or 30-days mortality (*P*< 0.01). This pattern was consistent in patients with severe sepsis or septic shock, even in the ICU or ED (all, *P*< 0.05).

Because sepsis is not a simple infectious disease but rather an aberrant host response with complex inflammatory pathophysiologic processes triggered by infection, no single pathogen or inflammatory biomarker has been enough to explain sepsis mortality. Clinically, three inflammatory biomarkers have been applied in patients with sepsis: C-reactive protein (CRP), procalcitonin, and presepsin. The major drawback of CRP is lack of specificity, and ultimately prognostication in sepsis is controversial [31, 32]; on the other hand, Procalcitonin has clearly showed mortality prediction value in meta-analysis [33, 34]. Presepsin, however, is a relatively new sepsis biomarker with no prior meta-analysis of its prognostic value; our meta-analysis assessed its mortality prediction in 10 studies with 1617 cases. There was a significant overall heterogeneity (*I*^2^ = 79%), and subsequent subgroup analysis divided by sepsis severity or conduction site could not resolve it, so we analyzed all of the sub-groups with the random effects model. In terms of the measurement method, it was homogeneous: all 10 studies used just the same PATHFAST kit. The cutoff values might possibly be generalized, but a wide range of cutoffs in various clinical situations (Table 2) makes this difficult. For kinetics, the peak presepsin concentration was displayed on day 1–3 of sepsis diagnosis [18]. Due to early elevation, in our study, the first sampling (within 24 hours) of presepsin might be an effective sepsis biomarker to predict mortality. It can be a good candidate for the multi-biomarker approach to timely prediction of sepsis mortality [21].

Regarding the sepsis definition, 8 of 10 studies used the previous definition [1, 24–27]; only two studies [18, 21] used the third international consensus definitions of sepsis and septic shock [1]. According to the new guideline [1], SIRS with infection is not sepsis anymore—organ dysfunction should be counted by an increase of SOFA score of 2 points or more correspond to severe sepsis in previous definitions. Therefore, we could say that the current 3^rd^ definition sepsis group (Fig 4, group I) also demonstrated significantly higher presepsin levels in non-survivors compared with survivors (*P*< 0.01). The degree of SMD tends to be modest in the severe group (Fig 4. group I or Fig 5. group I) as compared with mixed severity group (Fig 4 group II or Fig 5 group II), because the later groups possibly contain low presepsin levels in patients with infection-positive SIRS without organ dysfunction. A very high high mortality rate (72% in 6 months follow-up) was observed in the study that exclusively analyzed patients with septic shock [14].

Presepsin also has been shown to have prognostic value for situations other than sepsis such as cardiac surgery [35], hemophagocytic syndrome [36], or renal failure [37]. In particular, presepsin levels depend on renal function due to a 13-kDa small protein being filtered by the kidney [8, 38]. In our included studies, only half of them exclude comorbidities that might influence presepsin levels, which involves some possible confounding bias. But in real world, renal dysfunction is one of the sepsis-related organ failures and co-morbidities are not always clearly separated.

We acknowledge limitations to our systematic review and meta-analysis that are inherent in the data availability. First, not all the studies are prospective designs addressing concerns on selection bias that affect study quality. However, except for one case-control study, the original registries, even if retrospective in nature, were prospectively collected for sepsis and conducted with no interference in the routine clinical practice. Second, the authors’ efforts to contact the original article authors to get missing information were not as successful as hoped so this meta-analysis information depends on already-published data. Finally, there is possibly a publication bias against smaller and non-positive studies as suggested by a visual inspection of the funnel plot. For generalization of these meta-analysis results, greater access to original missing data or a larger number of prospective consecutive studies might be warranted.

In conclusion, first day presepsin levels had prognostic value to predict mortality in adult patients with sepsis, especially to predict in-hospital or 30-day mortality, regardless of sepsis severity or study location. Further controlled research is warranted for unified clinical information.

## Supporting information

S1 Checklist Prisma checklist. (DOC)

S1 Table Details of the quality assessment for each study. (DOCX)
