# Supplementary material for: Prognostic value of presepsin in adult patients with sepsis: Systematic review and meta-analysis
Source: PLoS One. 2018 Jan 24;13(1):e0191486. doi: 10.1371/journal.pone.0191486 (PMC5783380; doi:10.1371/journal.pone.0191486)
Supplement: S1 Table — (DOCX) [file pone.0191486.s002.docx]

| **4 criteria** | (1) the study included consecutive patients (selection bias); (2) the professionals who influenced the outcomes were blinded to the presepsin result at study entry (confusion bias); (3) the timing of blood sampling within 24 hours after admission (information bias); (4) the study excluded comorbidities potentially influencing presepsin levels and accuracy (confounding bias). | | | |
| --- | --- | --- | --- | --- |
| Study | Consecutive patients | Blinded to the result | Timing of blood sampling ≤ 24 hours | Excluding comorbidities |
| Liu et al. [13] | Yes | No | Yes | Yes |
| Behnes et al. [14] | No | Yes | Yes | Yes |
| Masson et al. [15] | No | No | Yes | No |
| Beňovská et al. [16] | No | No | Yes | Yes |
| Carpio et al. [17] | Yes | No | Yes | No |
| Ali et al. [18] | No | No | Yes | No |
| Klouche et al. [19] | Yes | Yes | Yes | Yes |
| El-Shafie et al. [20] | No | Yes | Yes | Yes |
| Kim et al. [21] | Yes | No | Yes | No |
| Yu et al. [22] | No | No | Yes | Yes |
